# Sodium content of restaurant dishes in China: a cross-sectional survey

**DOI:** 10.1186/s12937-022-00762-4

**Published:** 2022-02-17

**Authors:** Wenwen Du, Huijun Wang, Jiguo Zhang, Xiaofan Zhang, Nan Wei, Yuan Li, Monique Tan, Puhong Zhang, Feng J. He

**Affiliations:** 1 The National Institute for Nutrition and Health, Chinese Center for Disease Control and Prevention, 29 Nanwei Rd, Beijing, China; 2 The George Institute for Global Health at Peking University Health Science Center, Room 011, Unit 2, Tayuan Diplomatic Office Building No. 14 Liangmahe Nan Lu, Beijing, China; 3 Wolfson Institute of Preventive Medicine, Barts and The London School of Medicine & Dentistry, Queen Mary University of London, Charterhouse Square, EC1M-6BQ, London, UK

**Keywords:** Sodium, Restaurant, China

## Abstract

**Background:**

Sodium intake in China is extremely high and eating in restaurants is increasingly popular. Little research has explored the sodium level of restaurant dishes. The present study aims to assess the content and sources of sodium in Chinese restaurants.

**Methods:**

Cross-sectional data were obtained from the baseline survey of the Restaurant-based Intervention Study (RIS) in 2019. A total of 8131 best-selling restaurant dishes with detailed recipes from 192 restaurants in China were included. Sodium content per 100 g and per serving were calculated according to the Chinese Food Composition Table. The proportion of restaurant dishes exceeding the daily sodium reference intake level in a single serving and the major sources of sodium were determined.

**Results:**

Median sodium content in restaurant dishes were 487.3 mg per 100 g, 3.4 mg per kcal, and 2543.7 mg per serving. For a single serving, 74.9% of the dishes exceeded the Chinese adults’ daily adequate intake for sodium (AI, 1500 mg per day), and 62.6% of dishes exceeded the proposed intake for preventing non-communicable chronic diseases (PI, 2000 mg per day). Cooking salt was the leading source of sodium in Chinese restaurant dishes (45.8%), followed by monosodium glutamate (17.5%), food ingredients (17.1%), soy sauce (9.4%), and other condiments/seasonings (10.2%). More types of salted condiments/seasonings use were related to higher sodium level.

**Conclusions:**

The sodium levels in Chinese restaurant dishes are extremely high and variable. In addition to cooking salt, other salted condiments/seasonings also contribute a large proportion of sodium. Coordinated sodium reduction initiatives targeting the main sources of sodium in restaurant dishes are urgently needed.

## Background

High sodium intake is a public health concern worldwide, as it is linked to elevated blood pressure, which leads to cardiovascular disease [1–3]. In 2010, the global mean level of sodium intake was 3950 mg/day, nearly twice the World Health Organization (WHO) recommendation of 2000 mg/day [4, 5]. The Global Burden of Disease Study showed that 3 million deaths were attributable to the high salt intake in 2017 and about half of these deaths occurred in China [1]. East Asia is one of the regions with the highest sodium intake in the world [4]. According to the China National Nutrition and Health Surveillance (CNNHS) 2010–2012, Chinese adults consume 5013 mg/day of sodium on average, much higher than both the WHO and Chinese recommendations [6, 7]. High sodium intake is the leading risk factor for cardiometabolic mortality in China (population attributable fraction (PAF) of 17.3%) [8]. To tackle the adverse effects of high sodium consumption, many countries have implemented salt reduction strategies in recent years [9–12].

A national target of 20% reduction in salt intake by 2030 has been proposed in China’s health development agenda “Healthy China 2030” [13]. Determining local sodium intake levels and the main dietary contributors to sodium intake is critical to develop an effective sodium reduction strategy. In high-income countries, where the majority of the sodium consumed comes from processed foods, efforts focus on reducing the amount of salt added by the food industry, through setting incrementally lower salt targets for specific food categories [14, 15]. In China, salt added when cooking/preparing food is the leading source of sodium (69.2% of sodium intake), followed by soy sauce (8.2%), processed foods (6.0%), and chicken essence (4.5%) [6]. However, the above findings were obtained from the national nutrition and health survey, which did not include salt and condiment use in foods consumed away from home, which may have resulted in an underestimate of the total sodium intake [16]. Restaurant dishes have a higher sodium level than home-made foods [16], thus eating out is reported to be associated with higher intake of sodium [17, 18]. Besides their high sodium content, restaurant foods also are characterized by the use of salt substitutes and flavor enhancers [19], indicating different sources of sodium compared with home-made dishes. With rapid urbanization and economic development, the contribution of restaurant foods to the population’s sodium intake is increasing [20]. To achieve the sodium reduction goals, considerable efforts should thus be taken to reduce the amount of sodium added to restaurant foods [21].

“Action on Salt China” (ASC) is a unit for salt reduction, established in June 2017, in collaboration with Queen Mary University of London in the United Kingdom, The George institute for Global Health in China, the Chinese Center for Disease Control and Prevention, and other key national organizations [22]. The ASC program consists of four randomized controlled trials (RCTs), targeting various sources of salt intake in China. As one of the RCTs in ASC, the restaurant-based intervention study (RIS) is designed to test the feasibility and effectiveness of a package of interventions for salt reduction in 192 restaurants from 6 provinces of China. The present article describes the baseline data of RIS to determine the sodium content and sources in popular restaurant dishes in China, which will be helpful in developing effective strategies to reduce salt in restaurant foods.

## Methods

### Study design

The RIS baseline assessment survey was carried out in 6 provinces of China (Qinghai, Hebei, Heilongjiang, Hunan, Sichuan and Jiangxi) in May 2019. For each province, 2 counties of similar socioeconomic level were selected in the provincial capital city. Then, according to the selection criteria, 16 restaurants mainly offering Chinese cuisine were selected from each county, including 4 large-size, 8 medium-size and 4 small-size restaurants [23]. The detailed recipes of the 50 best-selling dishes from each restaurant were collected by trained investigators. For restaurants offering fewer than 50 dishes in their menu, all the dishes were included.

### Characteristics of restaurant dishes

To understand how sodium levels vary according to different characteristics of the restaurant dishes, we conducted our analyses by region, restaurant size and dish type. Region was defined as North (Qinghai, Hebei and Heilongjiang) and South (Hunan, Sichuan and Jiangxi). Restaurant size was classified into large (surface area > 500 m^2^ and ≤ 3000 m^2^, or number of seats > 250 and ≤ 1000), medium (surface area > 150 m^2^ and ≤ 500 m^2^, or number of seats > 75 and ≤ 250) and small (surface area ≤ 150 m^2^, or number of seats ≤75). Chinese cuisine substantially varied across geography areas, and was categorized into four types of dishes based on the cooking method: cold dish, fried dish, soup, and staples/snacks.

### Assessment of sodium content

A mobile-based electronic data collection system (EDC) developed by the Beijing University of Aeronautics and Astronautics was used for data collection. The recipes were collected by in-depth interviews with chefs who were familiar with the preparation and cooking of the dishes. The detailed recipes included the following information: name of the dish, edible percentage, ingredients and condiments/seasonings used (with amounts), dish type and cooking method. To improve the accuracy of the estimated amount of condiments/seasonings used, the investigators showed the weighed amount using a usual spoon or other measuring instruments during the interview. Sodium content of each dish was calculated according to the Chinese Food Composition Table, combining the sodium content of all ingredients and condiments/seasonings used for the dish. Sodium content is reported as sodium density (mg per 100 g, mg per kcal), as well as sodium (mg) per serving.

### Comparison to the Chinese dietary reference intakes (DRIs)

Sodium levels in a single serving of the restaurant dishes were compared to the Chinese DRIs: the daily AI (adequate intake, 1500 mg per day) and PI (proposed intakes for preventing non-communicable chronic diseases, 2000 mg per day) for adults aged 18 to 49 years old. Restaurant dishes were compared to the Chinese DRIs because restaurant sodium reduction targets have not been established in China. The proportions of restaurant dishes exceeding the daily sodium AI and PI in a single serving were calculated.

### Assessment of sodium sources

Based on the recipes, we classified the sources of sodium in restaurant dishes (according to the following categories: food ingredients, cooking salt, monosodium glutamate (including chicken powder and chicken essence), soy sauce, and other condiments/seasonings. We then estimated their respective contribution (in percentages) to sodium levels in dishes.

### Statistical analysis

We reported the median and interquartile range (IQR) for mg of sodium per 100 g, mg of sodium per kcal, and mg of sodium per serving in restaurant dishes, by region (North, South), restaurant size (large, medium and small), and dish type (cold dish, fried dish, soup and staples/snacks). Descriptive statistics for restaurant dishes, including %AI (sodium per serving divided by 1500 mg), and % of restaurant dishes exceeding the daily sodium AI and PI were conducted. The respective contributions of the main sources of sodium to total sodium content in restaurant dishes were calculated. As sodium values were not normally distributed, the non-parametric Wilcoxon signed-rank test was used to compare the median of sodium content among restaurant dishes stratified by area, restaurant size and dish type. We used SAS 9.4 (SAS Institute, Cary, NC) for data cleaning and analyses, and considered two-sided *P* < 0.05 as statistically significant.

## Results

### Sodium density and level in popular restaurant dishes in China

The analysis included a total 8131 dishes from 192 restaurants in 6 provinces of China. The sample encompassed 3829 (47.1%) and 4302 (52.9%) foods from the North and the South, and 2285 (28.1%), 4162 (51.2%) and 1684 (20.7%) foods from large, medium and small restaurants, respectively. The main dish type was fried dish (83.9%), followed by cold dish (11.7%), soup (3.0%) and staples/snacks (1.4%).

Overall, the restaurant dishes contained on average 487.3 (IQR: 291.1, 781.9) mg sodium per 100 g. Sodium levels varied significantly by area, restaurant size, and dish type (Table 1). The highest sodium densities (in mg per 100 g) were found in dishes from the South (566.3 mg per 100 g), in medium (497.5 mg per 100 g) and small (491.3 mg per 100 g) restaurants, and in soups (687.0 mg per 100 g), cold dishes (528.4 mg per 100 g) and fried dishes (480.9 mg per 100 g). Sodium density in mg per kcal showed similar trends, with an average of 3.4 (1.9, 6.4) mg of sodium per kcal. The dishes with the highest sodium density per kcal were from the South (3.6 mg), from medium (3.5 mg) and large (3.4 mg) restaurants, and in cold dishes (4.8 mg), soups (4.3 mg) and fried dishes (3.3 mg).

**Table 1:** Sodium levels in Chinese restaurant dishes

	N	Sodium (mg) per 100 g				Sodium (mg) per kcal			
		Median	P25	P75	***P*** value	Median	P25	P75	***P*** value
**Region**									
North	3829	415.7	250.7	682.8	< 0.0001	3.2	1.7	6.1	< 0.0001
South	4302	566.3	337.9	858.4		3.6	2.0	6.5	
**Restaurant size**									
Large	2285	466.1	278.1	763.3	0.0286	3.4	1.8	6.7	0.0006
Medium	4162	497.5	297.7	798.0		3.5	1.9	6.5	
Small	1684	491.3	302.1	767.1		3.1	1.8	5.7	
**Dish type**									
Cold dish	950	528.4	292.1	898.4	< 0.0001	4.8	2.1	10.3	< 0.0001
Fried dish	6819	480.9	291.1	762.3		3.3	1.9	6.0	
Soup	246	687.0	409.6	1143.3		4.3	2.3	9.3	
Staples/snacks	116	278.4	40.1	541.6		1.1	0.2	2.3	
**Total**	8131	487.3	291.1	781.9		3.4	1.9	6.4	

### Sodium levels per serving of restaurant dishes and comparison with Chinese dietary reference intakes

Table 2 shows serving size, sodium (mg) per serving, %AI, and proportions of restaurant dishes exceeding the daily sodium AI (1500 mg) and PI (2000 mg) by categories. The average serving size was 575.6 ± 318.0 g, providing 3331.2 ± 4156.9 mg sodium per serving, which represents 222.1% of the AI. Overall, 74.9% of the restaurant dishes exceeded Chinese adults’ daily sodium AI, and 62.6% of the dishes exceeded the adults’ PI. Dishes from the North, from large and medium restaurants, and belonging to the categories of fried dishes and soups had larger serving sizes and higher sodium levels (mg) per serving. In some instances, the higher sodium level per serving was mainly due to higher sodium density, such as in the South, in small restaurants and in cold dishes, while in other instances, it was due to larger serving sizes, such as in the North, in large restaurants, and in staples/snacks, or due to the combination of both serving size and sodium density, such as in medium restaurants, in fried dishes and soups.

**Table 2:** Sodium levels per serving of restaurant dishes in China, compared to the dietary reference intakes (DRIs)

	N	Mean serving size (g)		Sodium (mg) levels per serving						% of dishes exceeding the sodium DRIs levels ^b^	
		Mean	SD	Mean	SD	%AI ^a^	Median	P25	P75	AI: 1500 mg	PI: 2000 mg
**Region**											
North	3829	620.0	340.2	3270.2	4849.9	218.0	2345.5	1409.3	3734.5	72.1	58.9
South	4302	536.1	291.2	3385.5	3423.7	225.7	2759.3	1596.3	4220.1	77.4	65.9
**Restaurant size**											
Large	2285	611.4	377.9	3447.0	4774.4	229.8	2604.3	1527.5	4040.7	75.7	63.8
Medium	4162	576.4	291.3	3397.6	4219.8	226.5	2642.6	1579.3	4081.5	76.7	64.1
Small	1684	525.0	283.6	3010.1	2894.8	200.7	2283.6	1368.8	3901.9	69.5	57.4
**Dish type**											
Cold dish	950	426.6	237.2	2975.5	4540.7	198.4	2018.3	1091.0	3251.5	62.3	50.5
Fried dish	6819	598.9	317.3	3356.1	3887.3	223.7	2614.1	1575.8	4082.2	76.9	64.4
Soup	246	574.0	357.3	4805.4	8190.7	320.4	3373.0	2112.2	4852.2	85.8	77.6
Staples/snacks	116	431.3	454.6	1653.1	2011.6	110.2	934.7	107.6	2354.3	36.2	27.6
**Total**	8131	575.6	318.0	3331.2	4156.9	222.1	2543.7	1496.9	4030.9	74.9	62.6

*AI* Adequate intake, *DRI* Dietary reference intakes, *PI, PI-NCD* Proposed intake for preventing non-communicable chronic diseases

^a^Mean sodium levels per serving, expressed as a percentage of the daily AI for adults (1500 mg per day)

^b^Percentage of dishes in the groups that exceed the daily AI (1500 mg per day) or PI (2000 mg per day) per serving

### Sodium sources in restaurant dishes in China

**Fig. 1:**
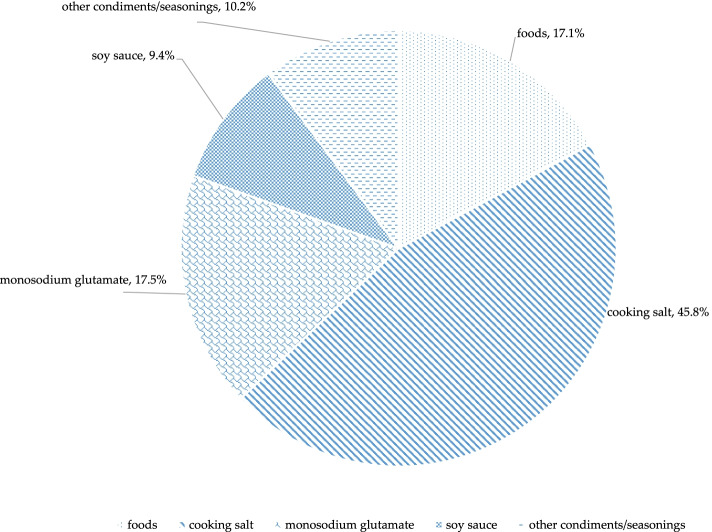
Sodium sources of restaurant dishes in China

Overall, cooking salt was the leading source of sodium in Chinese restaurant dishes, accounting for 45.8% of the sodium (Fig. 1). Monosodium glutamate was the second contributor (17.5%), followed by food ingredients (17.1%), soy sauce (9.4%), and other condiments/seasonings (10.2%). Beside the sodium already contained in the food ingredients, the majority (82.9%) of the sodium came from the use of salted condiments/seasonings added while cooking. However, cooking salt only contributed to less than half of the sodium contained in restaurant dishes. Monosodium glutamate, soy sauce and other condiments/seasonings (such as other sauces and compound condiments/seasonings) contributed to more than a third of total sodium content in restaurant dishes.

### Condiments/seasonings use and association with sodium level in Chinese restaurant dishes

**Fig. 2:**
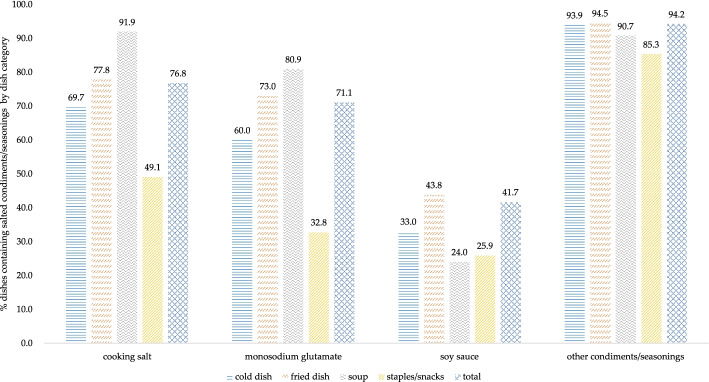
Percentage of restaurant dishes containing salted condiments/−seasonings by dish category in China

Figure 2 shows the proportion of restaurant dishes containing the main sources of sodium, by dish type. Overall, 76.8% of the dishes contained cooking salt, 71.1% contained monosodium glutamate and 41.7% contained soy sauce. Other condiments/seasonings were found in most (94.2%) restaurant dishes. Cooking salt was a major source of sodium in all type restaurant dishes, especially in soups (91.9%) and fried dishes (77.8%). Monosodium glutamate was more often used in soups (80.9%) and fried dishes (73.0%), followed by cold dishes (60.0%). Soy sauce was found in 43.8% of the fried dishes and 33.0% of the cold dishes. In staples/snack, prevalence of condiments/seasonings was below 50%, except for that of other condiments/seasonings.

Among all dishes included, nearly half (45.1%) contained 3 types of salted condiments/seasonings, followed by those containing 2 types (24.2%) and those containing 4 types (23.3%) condiments/seasonings (Table 3). Only 7.4% of foods only contained 1 type of salted condiments/seasonings. Dishes from the South, from small restaurants and categorized as fried dishes were more likely to contain all 4 types of salted condiments/seasonings. Generally, foods that contained more types of condiments/seasonings were significantly more likely to have a higher sodium level than those that contained fewer types of salted condiments/seasonings. This trend was seen in all subgroups, and the difference was significant (*P* < 0.0001). The median sodium level (mg) per 100 g in foods with 4 types of condiments/seasonings (644.4 mg per 100 g) was 4.8 times that of foods with 1 type of condiments/seasonings (134.8 mg per 100 g), 1.7 times of those with 2 types (380.7 mg per 100 g), 1.3 times of those with 3 types (506.4 mg per 100 g).

**Table 3:** Sodium level in Chinese restaurant dishes by types of salted condiment/seasonings used(*N* = 8131)

	***P*** value^*^	Number of salted condiment/seasonings types used^a^															
		1 type used				2 types used				3 types used				4 types used			
		%	sodium (mg) per 100 g			%	sodium (mg) per 100 g			%	sodium (mg) per 100 g			%	sodium (mg) per 100 g		
			Median	P25	P75		Median	P25	P75		Median	P25	P75		Median	P25	P75
**Region**																	
North	< 0.0001	8.8	113.2	29.9	396.4	22.0	342.1	191.9	539.2	49.1	445.5	290.8	713.2	20.1	500.6	346.6	783.8
South		6.2	146.2	43.9	488.8	26.1	408.0	259.6	705.6	41.6	578.9	370.1	911.0	26.1	704.3	533.2	998.9
**Restaurant size**																	
Large	< 0.0001	8.9	138.2	38.0	432.0	21.9	344.0	214.6	538.1	45.4	481.2	303.4	797.1	23.8	648.7	429.7	917.5
Medium		7.3	121.7	30.6	455.9	24.9	399.2	235.3	646.2	45.2	527.6	335.5	827.9	22.7	644.4	432.7	934.0
Small		5.9	138.7	35.6	405.8	25.7	386.2	234.5	666.3	44.5	486.7	317.0	763.0	24.0	640.3	444.8	901.4
**Dish type**																	
Cold dish	< 0.0001	15.1	139.4	36.0	479.9	25.1	470.2	272.9	709.2	47.3	615.4	372.4	970.3	12.6	729.6	414.6	1191.6
Fried dish		5.8	145.1	48.6	456.6	24.3	367.4	221.8	598.5	44.9	486.7	313.3	764.4	25.1	637.8	432.8	898.5
Soup		5.3	73.7	21.4	309.7	22.0	667.0	401.1	1141.9	52.4	696.4	388.2	1239.0	20.3	791.3	572.8	1118.6
Staples/snacks		48.3	39.5	5.3	178.1	14.7	393.0	276.7	554.7	24.1	507.0	360.8	777.6	12.9	461.1	301.8	777.4
**Total**	< 0.0001	7.4	134.8	35.9	441.6	24.2	380.7	228.3	630.7	45.1	506.4	321.6	810.0	23.3	644.4	436.6	919.7

^a^Types of salted condiments/seasonings include: cooking salt, monosodium glutamate, soy sauce, and other condiments/seasoning

^*^ Non-parametric Wilcoxon signed-rank tests of the sodium levels between different category amounts groups of salted condiment/seasonings

## Discussion

Commercially processed and restaurant foods are the main contributors for sodium intake in most high-income countries, e.g. the UK, USA [14, 24]. Although most sodium consumed in the Chinese population still mainly comes from salt and other salted condiments/seasonings added during cooking [6], there has been a rapid increase in the consumption of foods outside the home in the past decades. As such, reducing sodium in the out-of-home sector plays in increasingly important role for China to achieve the salt reduction targets by 2030.

### Main findings

Taking into account both the high sodium level in restaurant dishes and the increasing popularity of eating out, restaurant dishes are becoming increasingly important contributors to sodium intake in China. However, data on the sodium content of restaurant dishes in China is limited. The current study describes the sodium density and sodium content per serving, as well as sources of sodium in 8131 popular restaurant dishes from six provinces in China. Our results show that both the average level of sodium per 100 g and sodium level per serving are extremely high in Chinese restaurant dishes, with significant variations by region, restaurant size and dish type. On average, a single serving of a restaurant dish provided almost 2.2 times the daily recommended AI for sodium for Chinese adults. Sodium levels per serving in 74.9 and 62.6% of restaurant dishes exceeded the Chinese daily recommended AI (1500 mg) and PI (2000 mg), respectively.

### Comparison with other studies

Our findings of the very high and wide-ranging sodium levels in restaurant dishes are in agreement with those reported in several other studies [25–28]. Such high sodium levels of restaurant foods are attributed to either large serving sizes or high sodium density, or the combination of both depending on dish type [25]. In our study, the higher sodium level per serving in restaurant dishes, in some instances, was mainly due to higher sodium density, such as in the South, in small restaurants, and in cold dishes, while in other instances, it was mainly due to larger serving sizes, such as in the North, in large restaurants, and in staples/snacks, or due to the combination of both serving size and sodium density, such as in medium restaurants, and in fried dishes and soups. These differences may imply that specific sodium reduction strategies for restaurant dishes are needed in different situations.

Widespread use of salted condiments/seasonings is another explanation for the high sodium content in restaurant dishes. In leading Canadian chain restaurants, more than 60% of the dishes contained a salt substitute/enhancer, such as yeast extracts, calcium chloride, monosodium glutamate and potassium chloride [19]. In our study, salted condiments/seasonings contributed to 82.9% of the sodium found in restaurant dishes (37.1% if excluding cooking salt). This makes sodium sources in Chinese restaurant dishes more complex and diversified. With increasing development of compound condiments/seasonings, restaurant chefs prefer to add several kinds of flavorings rather than cooking salt only. We observed that more than two thirds of the restaurant dishes contained three or four types of salted condiments/seasonings. Sodium density of the dishes increased as more types of condiments/seasonings were used due to most of these condiments being produced by the food industry, effective sodium reduction in restaurants will also require cooperation with food manufacturers [29, 30].

### Implications

Many countries have implemented national or regional initiatives for sodium reduction in restaurants [9, 31–35], mainly including: menu labelling, setting sodium targets by food category, reformulation, raising consumer awareness, chef training, and promotional materials delivery. However, there are many potential barriers to reducing sodium content in restaurant dishes. The nutritional monitoring of restaurant dishes has shown that sodium levels continue to be high and the changes in sodium levels vary by food categories, with reductions only seen in a minority of the sampled foods [26, 27]. More effective sodium reduction strategies with multi-stakeholders’ cooperation are needed.

In China, cooking habits and consumers’ preferred taste make sodium reduction difficult in restaurant foods. However, attempts to explore effective sodium reduction strategies for restaurant in China have been made [36]. The RIS program, part of Action on Salt China (ASC), aims to determine the sodium level of restaurant dishes and evaluate the effectiveness of a comprehensive restaurant salt reduction package in China, which consists of menu labelling, chef and waiter/waitress training, reformulation, supportive environment building, and sodium reduction campaign. In addition, monitoring the sodium content of restaurant foods is essential to set specific sodium reduction targets by food categories and help consumers understand the benefits of opting for low-sodium options when eating out.

### Strengths and limitations

The strengths of our study include the large sample of restaurant dishes based on both major types of food served in the restaurants and foods frequently ordered by consumers from 6 provinces in China. The notable differences of restaurant food types between China and other countries [37, 38], call for more data based on local studies. Furthermore, we used standard reporting formats (sodium mg per 100 g) to facilitate comparisons across regions, restaurant sizes and dish types. We also reported the sodium content per serving, to help customers compare sodium content between various menu options. Finally, the large sample size in our study could capture the variability in sodium level in restaurant dishes, making the results more robust.

A number of limitations also exist. Sodium content assessing methods usually include laboratory analysis, menu labelling or online nutrition information provided by restaurant companies, and analysis with a nutrient database [39]. There are assessment differences between laboratory and menu items analysis, which may be due to differences in reported versus actual portion size and recall bias by chefs. However, due to the lack of publicly available menu nutrient values and the expensive cost of laboratory analyses, we considered menu items analysis the most cost-effective method to assess the sodium content of restaurant foods in China, especially with such a large sample size. In addition, with the rapid pace of restaurant foods development, this cross-sectional survey could not capture changes of sodium levels. Furthermore, the results in this study could not represent the sodium level for all Chinese restaurant dishes due to wide variations in restaurant foods. Some countries have tracking the sodium content in restaurant foods [40], which will provide dynamic data to guide restaurants in increasing the availability of lower-sodium foods and help consumers decrease their sodium intake.

## Conclusions

In conclusion, our study shows that the sodium content of the majority of popular restaurant dishes in China are extremely high and variable. Further, the large number of restaurant dishes that exceeded the daily AI and PI in a single serving, along with the widespread use of salted condiments/seasonings, demonstrate the need for a Chinese sodium reduction strategy that addresses all the major sodium sources. Coordinated government-led efforts should be implemented, with the participation of restaurants, food manufacturers, and consumers to reduce sodium level in restaurant foods, raise sodium-reduction awareness and ultimately lower population sodium intake.

## Data Availability

The datasets generated and/or analysed during the current study are not publicly available due to considerations of intellectual property. However, they may be available from the corresponding author on reasonable request.

## Abbreviations

RIS: Restaurant-based Intervention Study
AI: Adequate Intake
PI: Proposed Intake for Preventing Non-Communicable Chronic Diseases
CNNHS: China National Nutrition and Health Surveillance
PAF: Population Attributable Fraction
ASC: Action on Salt China
RCT: Randomized Controlled Trial
EDC: Electronic Data Collection System
DRIs: Dietary Reference Intakes
